# Supplementary material for: Fast and Sensitive Quantification of AccQ-Tag Derivatized Amino Acids and Biogenic Amines by UHPLC-UV Analysis from Complex Biological Samples
Source: Metabolites. 2022 Mar 21;12(3):272. doi: 10.3390/metabo12030272 (PMC8949038; doi:10.3390/metabo12030272)
Supplement: Supplementary file 1 [file metabolites-12-00272-s001.zip › Supplementary files/Supplementary figure caption.pdf]

Figure S1: Examination of sample loss after purification. The y axis show the mean peak area of the corresponding amino acid determined from two technical replicates.
