# Supplementary material for: Fast and Sensitive Quantification of AccQ-Tag Derivatized Amino Acids and Biogenic Amines by UHPLC-UV Analysis from Complex Biological Samples
Source: Metabolites. 2022 Mar 21;12(3):272. doi: 10.3390/metabo12030272 (PMC8949038; doi:10.3390/metabo12030272)
Supplement: Supplementary file 1 [file metabolites-12-00272-s001.zip › Supplementary files/Supplementary tables.pdf]

Table S1: Calibration parameters of the analyzed molecules in different matrices obtained with LC analysis. t_R: retention time, MQ: MilliQ water, LOD: limit of detection, LOQ: limit of quantification, R^2: goodness of fit.

Compound	Abbrev.	t_R (min)			LOD (μmol/L)			LOQ (μmol/L)			Linear range (μmol/L)			R^2			Calibration equation		
		MQ	Serum	Tears	MQ	Serum	Tears	MQ	Serum	Tears	MQ	Serum	Tears	MQ	Serum	Tears	MQ	Serum	Tears
Histidine	His	1.445	1.445	1.447	1.81	2.30	0.73	6.04	7.68	2.42	1.00-25.00	2.50-30.00	1.00-25.00	0.9960	0.9929	0.9962	y=3860.5x-2913	y=3892.1x-5107.1	y=3669.8x-2107.1
Asparagine	Asn	1.678	1.677	1.679	0.38	0.34	0.59	1.27	1.12	1.98	0.50-25.00	0.50-30.00	0.50-20.00	0.9946	0.9979	0.9981	y=3828.2x-190.91	y=3748x-663.53	y=3720.2x-818.48
Taurine	Tau	1.916	1.917	1.915	0.20	0.20	0.22	0.68	0.66	0.73	0.25-25.00	2.50-30.00	0.50-25.00	0.9987	0.9996	0.9977	y=4890x-523.91	y=4729.1x-2402.4	y=4420.8x+312.95
Serine	Ser	2.370	2.367	2.372	0.21	0.22	0.18	0.69	0.74	0.59	0.25-25.00	2.50-25.00	0.50-25.00	0.9963	0.9983	0.9938	y=4479.2x-66.304	y=4448.8x-1539.6	y=4009.3x+1387.1
Glutamine	Gln	2.547	2.546	2.550	2.02	1.91	1.67	6.74	6.37	5.58	0.50-25.00	0.50-30.00	1.00-25.00	0.9937	0.9973	0.9954	y=1417.1x-106.37	y=1286x+1204.9	y=1389.2x+476.11
Arginine	Arg	2.643	2.643	2.650	1.37	0.10	0.78	4.57	0.33	2.60	0.50-20.00	0.50-25.00	1.00-25.00	0.9971	0.9909	0.9889	y=1608.9x-750.81	y=1412.1x-372.8	y=1731x-193.31
Histamine	Hsn	2.748	2.744	2.754	0.72	0.75	0.52	2.39	2.51	1.73	0.50-25.00	2.50-25.00	0.50-25.00	0.9964	0.9974	0.9950	y=2629.8x-81.772	y=2668.8x-2084.9	y=2577.5x-546.46
Glycine	Gly	2.911	2.906	2.913	0.41	0.38	0.74	1.36	1.26	2.45	0.50-25.00	2.50-30.00	1.00-25.00	0.9961	0.9992	0.9932	y=4152.6x+635.83	y=4139.6x-1428.2	y=3710.6x+566.8
Ethanolamine	Eth	3.208	3.205	3.212	0.25	0.22	0.28	0.84	0.74	0.93	0.50-25.00	2.50-30.00	0.25-15.00	0.9952	0.9989	0.9995	y=4658.6x-173.92	y=4606.2x-2136.7	y=4269.7x+269.24
Aspartate	Asp	3.482	3.475	3.481	0.25	0.22	0.20	0.84	0.74	0.68	0.50-25.00	0.50-30.00	0.50-20.00	0.9870	0.9964	0.9976	y=4180.9x+305.26	y=4188.1x-278.46	y=4263.2-1075.9
Methylamine	Mea	3.876	3.870	3.879	0.26	0.31	0.20	0.85	1.02	0.68	0.25-25.00	0.50-30.00	0.25-15.00	0.9885	0.9965	0.9991	y=4057.6x-149.51	y=4080x-1009.4	y=3915x-408.56
Glutamate	Glu	4.845	4.840	4.841	0.44	0.49	0.36	1.48	1.64	1.20	0.25-25.00	0.50-30.00	0.25-15.00	0.9900	0.9971	0.9985	y=3650.5x+159.46	y=3830.6x-290.2	y=3578.2x+69.564
Citrulline	Cit	4.940	4.938	4.938	0.37	0.44	0.28	1.24	1.46	0.92	0.25-25.00	2.50-30.00	0.25-15.00	0.9962	0.9989	0.9997	y=4024.1x+36.865	y=4088.8x-1881.8	y=3734.5x+223.58
Threonine	Thr	5.493	5.500	5.514	0.18	0.17	0.20	0.61	0.56	0.66	0.25-25.00	2.50-30.00	1.00-25.00	0.9962	0.9991	0.9923	y=4577x-189.99	y=4518.4x-1481.8	y=4097.2x+1032.5
Alanine	Ala	5.946	5.956	5.977	0.22	0.23	0.12	0.74	0.78	0.41	0.25-25.00	1.00-30.00	0.25-15.00	0.9910	0.9973	0.9989	y=4485.8x-301.83	y=4444.7x+401.51	y=4229.7x+46.267
Ethylamine	Eta	6.146	6.157	6.181	0.18	0.13	0.15	0.61	0.44	0.50	0.50-25.00	0.25-30.00	0.25-15.00	0.9809	0.9948	0.9982	y=4651.6x+1115.2	y=4698.4x-191.1	y=4616.2x-299.08
Proline	Pro	6.356	6.378	6.417	0.13	0.16	0.26	0.44	0.53	0.88	0.25-25.00	2.50-30.00	1.00-25.00	0.9948	0.9987	0.9916	y=4175.9x-37.55	y=4144.9x-1235.4	y=3756.1x+882.19
Ornithine	Orn	6.914	6.970	7.061	0.15	0.17	0.16	0.50	0.56	0.55	0.25-25.00	2.50-30.00	0.25-15.00	0.9892	0.9964	0.9987	y=7707.5x-40.697	y=7813.7x-2198.1	y=7290.7x+363.3
Cysteine	Cys	7.336	7.397	7.492	0.17	0.14	0.20	0.56	0.47	0.67	0.50-25.00	2.50-30.00	1.00-25.00	0.9982	0.9995	0.9974	y=3983x-579.2	y=3855x-1903.3	y=3483.4x+443.04
Lysine	Lys	7.521	7.578	7.668	0.23	0.26	0.52	0.75	0.85	1.74	0.25-25.00	2.50-30.00	0.25-15.00	0.9859	0.9945	0.9983	y=6416.8x+263.85	y=6297.1x-552.86	y=5433.3x-148.64
Tyrosine	Tyr	7.596	7.643	7.724	0.17	0.24	0.78	0.56	0.81	2.60	0.50-25.00	2.50-30.00	0.50-25.00	0.9990	0.9996	0.9991	y=4213.5x-194.44	y=3992.8x-2012.4	y=3402.4x+2.7552
Putrescine	Put	7.665	7.719	7.796	0.14	0.26	0.13	0.47	0.87	0.44	0.25-25.00	2.50-30.00	0.25-15.00	0.9891	0.9966	0.9987	y=7099.6x-194.6	y=7616.2x-3549.9	y=7072.7x-42.892
Methionine	Met	7.865	7.887	7.922	0.13	0.13	0.38	0.44	0.43	1.28	0.50-25.00	2.50-30.00	1.00-25.00	0.9970	0.9994	0.9949	y=4363.9x-142.2	y=4184x-1767.6	y=3639.9x+879.88
Serotonin	Stn	7.920	7.935	7.962	0.81	0.12	0.38	2.71	0.41	1.28	0.50-30.00	2.50-25.00	0.25-30.00	0.9980	0.9978	0.9981	y=3858.1x-1256.7	y=3782.5x-4345.7	y=3295.7x-47.009
Valine	Val	7.991	8.001	8.019	0.11	0.39	0.21	0.38	1.30	0.69	0.25-25.00	2.50-30.00	0.25-20.00	0.9943	0.9985	0.9982	y=3972.4x+114.77	y=3670.1x-486.45	y=2371.7x+142.08
Cadaverine	Cad	8.029	8.033	8.044	0.21	0.14	0.33	0.69	0.47	1.10	0.25-25.00	2.50-30.00	0.25-15.00	0.9858	0.9947	0.9977	y=6926.8x+255.02	y=7099x-2537.3	y=5684.9x-79.418
Tyramine	Tra	8.069	8.075	8.090	0.30	0.30	0.20	0.99	0.99	0.65	0.25-25.00	2.50-30.00	0.25-20.00	0.9983	0.9996	0.9992	y=4229.9x+82.921	y=4164.2x-1991	y=4183.3x-270.81
Isoleucine	Ile	8.446	8.453	8.468	0.13	0.17	0.18	0.43	0.55	0.61	0.25-25.00	2.50-30.00	0.50-20.00	0.9949	0.9986	0.9979	y=4553x-38.987	y=4547.8x-1589.8	y=4396.9x-967.46
Leucine	Leu	8.535	8.544	8.562	0.11	0.11	0.25	0.36	0.38	0.82	0.25-20.00	0.50-25.00	0.25-20.00	0.9982	0.9974	0.9988	y=5886.6x-913.74	y=5546x+747.22	y=5280.4x+157.72
Phenylalanine	Phe	8.662	8.673	8.696	0.17	0.16	0.21	0.55	0.53	0.70	0.25-25.00	2.50-30.00	0.50-25.00	0.9987	0.9996	0.9982	y=4783.6x-200.49	y=4600.5x-2403.9	y=4259x+40.601
Tryptophan	Trp	8.785	8.794	8.807	0.61	0.22	0.20	2.02	0.72	0.66	1.00-25.00	2.50-30.00	0.25-25.00	0.9992	0.9995	0.9993	y=3638.1x-1608.8	y=3438.7x-3021.5	y=3197.3x-322.9
Tryptamine + 2-phenethyl-amine	Tpa + Pha	8.932	8.931	8.933	0.14	0.18	0.27	0.47	0.58	0.89	0.25-25.00	2.50-30.00	0.25-25.00	0.9975	0.9995	0.9966	y=10129x-1079.1	y=9741.7x-5269.3	y=8947.3x+2112.5

Table S2: Calibration parameters of the analyzed molecules in different matrices obtained by SRM. t_R: retention time, MQ: MilliQ water, LOD: limit of detection, LOQ: limit of quantification, R^2: goodness of fit.

Compound	Abbrev.	LOD (µmol/L)			LOQ (µmol/L)			Linear range (µmol/L)			R^2			Calibration equation		
		MQ	Serum	Tears	MQ	Serum	Tears	MQ	Serum	Tears	MQ	Serum	Tears	MQ	Serum	Tears
Histidine	His	0.62	0.38	0.05	2.05	1.28	0.18	0.25-5.00	0.25-15.00	0.25-30.00	0.9501	0.9571	0.9901	y=12312x+188.41	y=6690.8x+299.92	y=5816x+583
Asparagine	Asn	0.10	0.07	0.09	0.35	0.22	0.31	0.25-25.00	0.25-30.00	0.25-30.00	0.9922	0.9971	0.9812	y=371420x+303930	y=333720x+42504	y=301810x+59681
Taurine	Tau	0.11	0.08	0.13	0.35	0.26	0.45	0.25-15.00	0.25-15.00	0.25-10.00	0.9844	0.9939	0.991	y=124750x+184720	y=1091100x+222630	y=1132800x+131900
Serine	Ser	0.34	0.12	0.16	1.13	0.41	0.54	0.25-20.00	0.25-15.00	0.25-20.00	0.9728	0.9939	0.9914	y=230160x+43664	y=188530x+35518	y=173870x+41266
Glutamine	Gln	0.27	0.11	0.10	0.89	0.38	0.35	0.25-20.00	0.25-30.00	0.25-20.00	0.9811	0.9933	0.9945	y=116290x+9123.6	y=92320x+76101	y=89145x+6316.2
Arginine	Arg	0.06	0.05	0.04	0.20	0.17	0.13	0.25-25.00	0.25-30.00	0.25-25.00	0.9969	0.9964	0.9958	y=75362x+1810.2	y=69439x+2550.3	y=64679x+4159.5
Histamine	Hsn	0.20	0.11	0.07	0.65	0.36	0.25	0.25-15.00	0.25-10.00	0.25-10.00	0.9718	0.9954	0.99	y=1628100x+32780	y=1475700x+176490	y=1527800x+228440
Glycine	Gly	0.40	0.14	0.10	1.33	0.47	0.35	0.25-15.00	0.25-15.00	0.25-20.00	0.969	0.9937	0.9928	y=87225x+43383	y=71687x+23415	y=66764x+22287
Ethanolamine	Eth	0.06	0.10	0.07	0.20	0.33	0.24	0.25-2.50	0.25-5.00	0.25-2.50	0.9946	0.9861	0.9891	y=7892700x+578120	y=5700900x+1002000	y=6852100x+596130
Aspartate	Asp	0.06	0.11	0.13	0.19	0.36	0.44	0.25-20.00	0.25-30.00	0.25-20.00	0.9957	0.9957	0.9945	y=243470x+15357	y=213400x+10136	y=209250x+14457
Methylamine	Mea	0.09	0.07	0.04	0.31	0.22	0.13	0.25-2.50	0.25-2.50	0.25-2.50	0.9899	0.9794	0.9879	y=10998000x+854830	Y=9605800X+780600	y=9828200x+1005600
Glutamate	Glu	0.18	0.16	0.05	0.59	0.52	0.16	0.25-20.00	0.25-30.00	0.25-20.00	0.9914	0.9955	0.9961	y=200980x+20420	y=172740x+15552	y=167000x+19499
Citrulline	Cit	0.11	0.05	0.13	0.36	0.18	0.43	0.25-20.00	0.25-30.00	0.25-20.00	0.9903	0.9895	0.9924	y=787570x+115960	y=651310x+115560	y=665430x+99481
Threonine	Thr	0.11	0.05	0.09	0.35	0.18	0.31	0.25-25.00	0.25-30.00	0.25-20.00	0.9911	0.999	0.9969	y=130650x+11749	y=114800x+15572	y=110240x+10328
Alanine	Ala	0.18	0.09	0.11	0.59	0.29	0.37	0.25-20.00	0.25-30.00	0.25-20.00	0.9931	0.9931	0.9963	y=321060x+38689	y=276410x+111620	y=282980x+38419
Ethylamine	Eta	0.20	0.20	0.15	0.66	0.67	0.48	0.25-1.00	0.25-1.00	0.25-1.00	0.9967	0.9876	0.996	y=3884500x+13176	y=12643000x+526520	y=11584000x+1455100
Proline	Pro	0.12	0.06	0.07	0.39	0.20	0.22	0.25-15.00	0.25-15.00	0.25-15.00	0.9897	0.9955	0.9729	y=910020x+106240	y=819010x+132710	y=788890x+140950
Ornithine	Orn	0.12	0.08	0.07	0.38	0.26	0.22	0.25-20.00	0.25-30.00	0.25-20.00	0.9943	0.9953	0.9981	y=336010x+41211	y=285230x+35028	y=287020x+39900
Cysteine	Cys	0.07	0.04	0.04	0.23	0.15	0.13	0.25-25.00	0.25-30.00	0.25-20.00	0.9953	0.9973	0.9977	y=245860x+19091	y=211030x+26068	y=214730x+14727
Lysine	Lys	0.07	0.08	0.07	0.32	0.27	0.25	0.25-25.00	0.25-30.00	0.25-20.00	0.9865	0.9933	0.9944	y=84038x+14540	y=70943x+16772	y=71621x+7238.7
Tyrosine	Tyr	0.11	0.09	0.08	0.38	0.31	0.25	0.25-25.00	0.25-30.00	0.25-20.00	0.9905	0.995	0.9956	y=154990x+18819	y=128700x+16351	y=130000x+14760
Putrescine	Put	0.10	0.04	0.08	0.32	0.12	0.26	0.25-20.00	0.25-20.00	0.25-20.00	0.9912	0.9951	0.9917	y=632210x+113100	y=569010x+77417	y=564250x+101050
Methionine	Met	0.09	0.03	0.07	0.29	0.10	0.23	0.25-15.00	0.25-20.00	0.25-20.00	0.9949	0.9946	0.9912	y=751150x+75080	y=571340x+64245	y=534400x+69109
Serotonin[1]	Stn[1]	0.08	0.03	0.12	0.25	0.10	0.39	0.25-5.00	0.25-5.00	0.25-7.50	0.9908	0.9992	0.9882	y=2161700x+160200	y=1782800x+78256	y=1620900x+222090
Serotonin[2]	Stn[2]	0.17	0.03	0.42	0.56	0.08	1.39	0.25-15.00	0.25-20.00	0.25-20.00	0.9904	0.9972	0.9694	y=31572x+2602.1	y=31062x+1033.9	y=23389x+2350.9
Valine	Val	0.09	0.05	0.11	0.28	0.15	0.37	0.25-15.00	0.25-10.00	0.25-20.00	0.989	0.9972	0.9873	y=736370x+102230	y=673670x+181120	y=567960x+102060
Cadaverine	Cad	0.13	0.05	0.09	0.42	0.15	0.29	0.25-15.00	0.25-10.00	0.25-20.00	0.984	0.9955	0.9913	y=689730x+152960	y=645020x+48681	y=547430x+106030
Tyramine	Tra	0.28	0.13	0.16	0.93	0.44	0.54	0.25-1.00	0.25-1.00	0.25-1.0	0.9649	0.9946	0.9782	y=7583600x+554690	y=6577500x+94653	y=6118600x+127410
Isoleucine	Ile	1.04	0.03	0.16	3.47	0.10	0.52	0.25-1.00	0.25-5.00	0.25-5.00	0.9925	0.9943	0.9747	y=3822900x-3670.2	y=2493000x+431770	y=2356600x+322130
Leucine	Leu	0.02	0.05	0.16	0.07	0.18	0.54	0.25-2.50	0.25-5.00	0.25-5.00	0.9927	0.9851	0.9771	y=3502400x+164690	y=2581300x+507180	y=2410800x+298750
Phenylalanine	Phe	0.10	0.05	1.57	0.32	0.15	5.25	0.25-15.00	0.25-30.00	0.25-20.00	0.996	0.9971	0.9818	y=503990x+35433	y=412360x+55367	y=400020x+35017
Tryptophan	Trp	0.10	0.04	0.41	0.33	0.13	1.37	0.25-15.00	0.25-30.00	0.25-20.00	0.9956	0.9936	0.9707	y=560190x+32549	y=432100x+45221	y=413050x+29569
Tryptamine	Tpa	0.60	0.67	0.55	2.00	2.25	1.85	0.25-2.50	0.25-2.50	0.25-2.50	0.9641	0.9432	0.9506	y=3685800x+385210	y=3700100x+106100	y=2748700x+354500
2-phenethyl-amine	Pha	0.07	0.86	0.73	0.24	2.86	2.42	0.25-2.50	0.25-1.00	0.25-2.50	0.9402	0.9947	0.9533	y=4511600x+1890500	y=5292600x+373080	y=5132000x+857750

Table S3: Intra- and interday accuracy and precision. RSD: relative standard deviation, QC: quality control.

Compound	QC concentration (μmol/L)	Intraday variability (n=5)									Interday variability (n=15)								
		Mean concentration (μmol/L)			RSD%			Accuracy%			Mean concentration (μmol/L)			RSD%			Accuracy%		
		MQ	Serum	Tears	MQ	Serum	Tears	MQ	Serum	Tears	MQ	Serum	Tears	MQ	Serum	Tears	MQ	Serum	Tears
Histidine	2.5	2.80	2.82	2.78	1.23	0.83	0.72	112.18	112.83	111.15	2.79	2.82	2.76	1.43	1.54	1.28	111.47	112.59	110.48
	7.5	8.13	7.98	8.12	1.99	2.18	0.13	108.41	106.41	108.25	7.96	7.89	7.99	2.07	1.73	2.32	106.12	105.14	106.59
	15	15.19	16.57	16.76	6.22	0.27	1.84	101.25	110.48	111.71	15.98	16.37	16.86	5.36	1.37	1.45	106.53	109.10	112.37
Asparagine	2.5	2.73	2.71	2.76	3.41	0.72	0.88	109.13	108.37	110.24	2.73	2.71	2.83	3.90	2.69	4.07	109.12	108.51	113.04
	7.5	8.20	8.49	8.36	2.30	0.71	0.50	109.32	113.26	111.50	8.38	8.58	8.28	2.35	2.68	1.87	111.71	114.35	110.36
	15	15.49	16.43	17.10	1.95	1.65	0.59	103.29	109.56	114.00	15.95	15.79	16.60	2.85	4.22	2.56	106.34	105.24	110.69
Taurine	2.5	2.40	2.73	2.40	2.62	0.76	3.35	95.89	109.36	96.00	2.37	2.79	2.45	2.40	3.15	2.98	94.75	111.51	97.83
	7.5	7.36	8.30	7.97	1.31	0.52	1.10	98.10	110.60	106.29	7.34	8.23	7.75	1.47	1.09	3.49	97.89	109.72	103.32
	15	13.96	16.09	16.21	0.73	0.20	1.25	93.05	107.24	108.09	14.42	15.41	16.17	2.47	4.57	0.91	96.15	102.74	107.79
Serine	2.5	2.57	2.68	2.56	1.84	1.01	2.35	102.84	107.09	102.27	2.56	2.69	2.60	2.37	1.08	1.93	102.29	107.51	103.93
	7.5	7.74	8.36	8.33	0.60	0.46	1.24	103.19	111.47	111.04	7.71	8.33	8.03	0.66	0.77	4.69	102.85	111.10	107.08
	15	14.19	15.73	17.12	0.24	0.24	0.42	94.62	104.89	114.13	14.82	15.04	16.70	3.15	4.55	1.94	98.82	100.25	111.33
Glutamine	2.5	2.73	2.64	2.73	1.38	3.12	0.88	109.27	105.43	109.06	2.68	2.58	2.66	2.19	4.54	3.29	107.04	103.32	106.48
	7.5	8.47	8.08	8.00	2.10	2.89	1.34	112.87	107.72	106.72	8.32	7.99	7.96	2.58	2.66	2.57	110.95	106.53	106.09
	15	16.48	16.31	16.63	1.98	1.43	1.66	109.84	108.74	110.86	16.56	16.33	16.33	1.74	2.38	3.29	110.42	108.84	108.88
Arginine	2.5	2.86	2.52	2.51	6.22	1.45	4.04	114.22	100.85	100.44	2.50	2.40	2.41	14.93	8.95	4.71	100.00	96.09	96.48
	7.5	8.31	7.44	7.57	3.10	2.47	4.06	110.76	99.24	100.94	7.42	7.32	6.86	9.36	7.39	14.98	98.97	97.64	91.51
	15	15.60	16.34	13.80	3.65	1.22	2.40	104.02	108.90	91.99	14.69	15.17	14.01	10.11	7.07	8.49	97.95	101.15	93.37
Histamine	2.5	2.25	2.61	2.41	2.34	0.37	2.58	89.82	104.26	96.26	2.13	2.59	2.26	7.99	1.80	8.50	85.36	103.65	90.46
	7.5	7.57	6.82	7.15	2.58	1.25	1.43	100.87	90.92	95.26	6.82	6.83	6.60	8.50	2.13	12.53	90.92	91.01	87.98
	15	13.47	14.41	13.71	2.01	1.60	2.34	89.77	96.03	91.39	13.44	13.37	14.00	2.79	6.16	3.79	89.63	89.15	93.36
Glycine	2.5	2.41	2.74	2.67	2.20	2.23	1.88	96.25	109.67	106.76	2.46	2.73	2.70	3.02	1.66	1.75	98.57	109.27	107.95
	7.5	7.58	8.48	8.52	0.92	1.56	0.66	101.12	113.03	113.54	7.57	8.44	8.21	1.13	1.09	4.63	100.86	112.57	109.49
	15	14.23	16.15	16.98	1.26	0.89	0.48	94.84	107.64	113.20	14.91	15.41	16.93	3.58	4.57	0.69	99.37	102.76	112.86
Ethanolamine	2.5	2.55	2.72	2.73	1.73	0.87	2.55	101.81	108.84	109.18	2.52	2.77	2.68	1.61	2.50	2.80	100.81	110.65	107.22
	7.5	7.53	8.21	8.21	0.93	1.01	1.08	100.34	109.50	109.41	7.55	8.16	7.85	1.05	0.85	4.73	100.60	108.74	104.67
	15	13.96	15.48	16.55	0.50	0.35	0.50	93.08	103.19	110.34	14.57	14.79	16.15	2.90	4.81	2.03	97.11	98.57	107.65
Aspartate	2.5	2.52	2.59	2.79	2.94	2.06	2.43	100.83	103.46	111.62	2.52	2.58	2.82	2.84	6.95	2.86	100.69	103.06	112.67
	7.5	7.90	8.42	8.07	1.10	1.04	0.65	105.28	112.27	107.60	7.94	8.18	7.72	0.87	2.33	4.23	105.80	109.00	102.86
	15	14.26	14.36	15.86	0.64	0.29	0.92	95.09	95.75	105.70	14.91	14.99	15.12	3.95	2.14	3.87	99.42	93.30	100.77
Methylamine	2.5	2.66	2.79	2.73	2.71	2.01	1.61	106.28	111.55	109.06	2.62	2.74	2.81	3.11	5.53	3.23	104.77	109.41	112.46
	7.5	7.96	8.49	8.33	1.47	0.64	1.53	106.13	113.21	111.05	8.03	8.27	7.87	1.64	2.35	4.85	107.08	110.20	104.88
	15	14.48	14.58	16.83	0.34	0.47	0.19	96.56	97.22	112.17	15.04	14.36	15.90	4.41	2.21	4.57	100.30	95.73	105.99

	2.5	2.76	2.67	2.77	5.09	0.53	1.16	110.43	106.71	110.84	2.72	2.73	2.83	4.03	3.97	3.07	108.86	109.14	113.33
Glutamate	7.5	8.22	8.46	8.27	4.64	2.21	0.63	109.56	112.84	110.24	8.31	8.53	8.09	2.78	1.80	3.07	110.83	113.76	107.88
	15	15.07	14.96	16.78	4.09	0.34	0.43	100.45	99.73	111.87	15.81	14.61	16.36	4.37	2.34	2.33	105.43	97.41	109.09
	2.5	2.67	2.79	2.78	4.33	0.87	0.37	106.61	111.50	111.17	2.66	2.78	2.84	2.82	1.62	1.85	106.43	111.30	113.58
Citrulline	7.5	8.12	8.50	8.60	3.63	0.62	0.41	108.21	113.33	114.63	8.13	8.58	8.40	2.09	0.82	3.91	108.42	114.34	112.04
	15	15.05	16.12	17.03	3.98	0.23	0.24	100.31	107.47	113.54	15.73	15.58	17.16	3.90	4.28	1.07	104.88	103.86	114.38
	2.5	2.60	2.67	2.64	2.03	0.25	1.02	103.95	106.65	105.41	2.57	2.73	2.65	2.23	3.82	1.11	102.88	109.32	105.86
Threonine	7.5	7.73	8.50	8.40	0.55	0.49	0.63	103.02	113.31	111.99	7.76	8.37	8.09	1.01	1.18	4.59	103.46	111.62	107.92
	15	14.26	15.81	17.22	0.28	0.41	0.45	95.07	105.40	114.80	14.89	15.06	16.76	3.16	4.67	2.11	99.30	100.40	111.72
	2.5	2.68	2.71	2.72	0.96	1.13	2.47	107.17	108.53	108.93	2.64	2.69	2.74	3.30	1.54	2.50	105.49	107.57	109.54
Alanine	7.5	7.96	8.17	8.47	0.27	1.00	0.38	106.18	108.95	112.86	8.03	8.42	8.01	0.97	2.22	5.85	107.01	112.23	106.75
	15	14.50	15.36	17.04	0.30	0.36	0.38	96.67	102.43	113.59	15.14	14.85	16.32	3.53	3.25	3.41	100.96	99.02	108.79
	2.5	2.61	2.76	2.82	2.68	1.59	1.34	104.50	110.33	112.98	2.57	2.68	2.84	2.00	6.32	2.09	102.84	107.29	113.42
Ethylamine	7.5	8.15	8.53	8.44	0.65	0.23	0.57	108.63	113.78	112.55	8.17	8.38	7.95	1.32	1.72	4.74	108.97	111.75	105.99
	15	14.89	14.52	16.99	0.17	0.26	0.25	99.28	96.81	113.26	15.41	14.42	15.86	5.19	1.27	5.64	102.74	96.11	105.75
	2.5	2.53	2.80	2.55	0.32	8.50	0.59	101.26	112.11	102.13	2.53	2.79	2.59	1.59	5.45	1.36	101.25	111.84	103.77
Proline	7.5	7.67	8.36	8.30	0.22	0.31	0.36	102.21	111.47	110.72	7.67	8.31	7.96	0.49	0.48	4.65	102.23	110.83	106.19
	15	14.25	15.65	17.05	1.55	1.26	0.21	95.02	104.35	113.64	14.77	14.95	16.53	2.81	4.49	2.33	98.44	99.63	110.22
	2.5	2.70	2.82	2.78	1.33	0.27	0.32	108.15	112.89	111.22	2.72	2.80	2.86	1.27	0.73	2.50	108.94	112.13	114.49
Ornithine	7.5	7.97	8.55	8.54	1.03	0.25	0.27	106.32	114.02	113.88	8.11	8.44	8.10	1.64	1.03	4.64	108.11	112.54	108.01
	15	14.39	15.04	17.01	0.37	0.20	0.46	95.94	100.26	113.43	15.21	14.49	16.35	4.45	3.97	3.39	101.37	96.60	108.99
	2.5	2.56	2.81	2.63	0.46	0.51	1.69	102.51	112.52	105.32	2.57	2.84	2.66	1.51	1.58	1.23	102.58	113.39	106.25
Cysteine	7.5	7.59	8.31	8.35	0.86	0.23	0.39	101.18	110.75	111.36	7.51	8.21	8.12	0.98	0.91	4.31	100.16	109.44	108.32
	15	14.16	16.20	17.16	0.27	0.23	0.35	94.40	108.00	114.40	14.59	15.22	16.77	2.23	6.29	1.85	97.30	101.46	111.83
	2.5	2.79	2.75	2.79	2.43	1.54	0.99	111.50	109.86	111.81	2.73	2.77	2.79	2.17	1.41	1.33	109.12	110.87	111.58
Lysine	7.5	8.39	8.25	8.41	0.62	1.12	0.61	111.87	110.02	112.14	8.27	8.29	8.42	2.36	0.83	0.89	110.32	110.53	112.31
	15	15.16	16.11	16.94	0.56	0.41	1.27	101.07	107.41	112.96	15.48	15.20	17.14	4.40	5.56	1.30	103.20	101.35	114.27
	2.5	2.49	2.72	2.64	2.37	1.47	1.37	99.44	108.79	105.61	2.36	2.72	2.62	4.47	0.92	1.73	94.30	108.60	104.60
Tyrosine	7.5	7.88	7.68	7.44	1.77	1.88	3.42	105.01	102.45	99.21	7.58	7.54	7.48	3.45	3.37	4.59	101.07	100.51	99.78
	15	15.40	15.92	16.00	0.74	1.42	3.36	102.68	106.12	106.67	15.14	15.26	16.12	2.03	3.85	3.98	100.90	101.70	107.47
	2.5	2.80	2.82	2.69	1.39	0.22	1.32	111.89	112.97	107.82	2.83	2.81	2.80	1.92	0.79	2.91	113.12	112.44	112.03
Putrescine	7.5	8.37	8.43	8.46	1.32	0.55	0.33	111.53	112.38	112.81	8.57	8.45	8.06	1.96	0.95	4.89	114.25	112.60	107.51
	15	15.21	14.97	17.14	0.51	0.70	0.69	101.40	99.77	114.26	16.19	14.68	16.54	4.79	3.04	2.85	107.92	97.87	110.23
	2.5	2.73	2.81	2.50	2.04	0.47	1.07	109.01	112.38	100.13	2.72	2.79	2.52	1.25	1.71	2.85	108.65	111.96	100.61
Methionine	7.5	8.17	8.03	8.29	0.74	0.84	0.82	108.96	107.08	110.56	8.07	8.04	8.39	1.55	1.11	2.52	107.59	107.16	111.91
	15	15.20	16.93	16.92	0.23	1.12	1.09	101.34	112.87	112.79	15.67	16.31	16.95	2.30	4.51	0.79	104.47	108.75	113.03
Serotonin	2.5	2.37	2.85	2.48	1.09	0.41	0.96	94.73	114.12	99.22	2.31	2.84	2.44	2.46	0.90	1.58	92.22	113.42	97.50
	7.5	6.71	7.60	7.48	0.28	0.16	0.31	89.52	101.34	99.78	6.48	7.53	7.26	3.63	1.14	3.76	86.45	100.37	96.85

	15	12.85	15.17	15.71	0.18	0.16	0.25	85.66	101.13	104.73	12.93	14.39	15.53	1.31	5.62	0.93	86.18	95.96	103.53
Valine	2.5	2.71	2.81	2.61	1.29	1.32	3.41	108.37	112.39	104.26	2.63	2.76	2.62	2.94	3.08	4.44	105.32	110.46	104.69
	7.5	8.37	8.20	8.29	1.30	1.38	2.58	111.55	109.36	110.50	7.93	8.14	8.22	4.35	1.57	1.80	105.74	108.57	109.54
	15	15.77	16.84	16.81	0.14	1.17	1.97	105.15	112.23	112.07	15.67	16.16	16.20	1.65	4.15	3.14	104.49	107.70	108.02
Cadaverine	2.5	2.79	2.74	2.76	3.24	0.84	3.23	111.39	109.57	110.22	2.77	2.78	2.77	3.41	2.80	2.36	110.89	111.12	110.76
	7.5	8.45	8.55	8.35	1.11	0.14	1.76	112.68	113.98	111.39	8.58	8.61	8.39	1.57	1.43	1.59	114.41	114.84	111.84
	15	15.36	15.69	17.02	0.72	0.19	1.40	102.39	104.63	113.45	16.33	15.37	17.05	5.60	3.75	0.99	108.89	102.44	113.68
Tyramine	2.5	2.54	2.69	2.69	0.72	1.91	0.38	101.59	107.66	107.94	2.66	2.73	2.83	3.41	1.68	3.39	106.48	109.03	113.15
	7.5	7.68	8.40	7.85	0.81	0.28	0.65	102.33	112.00	104.61	7.91	8.52	7.96	2.30	2.55	3.70	105.52	113.62	106.10
	15	14.64	16.61	15.79	0.63	0.10	0.33	97.61	110.71	105.27	15.56	16.41	16.23	4.37	2.84	2.09	103.75	109.39	108.20
Isoleucine	2.5	2.52	2.77	2.82	1.10	5.54	1.17	100.81	110.97	112.67	2.56	2.7	2.75	2.32	3.22	2.27	102.47	110.86	109.95
	7.5	7.54	8.27	8.14	1.44	1.14	1.35	100.58	110.23	108.48	7.72	8.29	7.95	1.89	0.73	4.02	102.95	110.59	105.95
	15	13.82	15.34	16.29	1.76	1.18	1.31	92.13	102.29	108.61	14.78	14.89	16.03	4.89	3.62	1.47	98.52	99.24	106.85
Leucine	2.5	2.74	2.67	2.75	1.70	0.94	0.76	109.68	106.67	109.99	2.74	2.62	2.79	2.70	5.32	1.26	109.74	104.90	111.65
	7.5	7.73	8.26	8.39	0.46	0.31	0.48	103.09	110.17	111.81	7.83	8.15	7.53	1.32	1.02	14.45	104.40	108.69	100.36
	15	13.87	15.02	16.46	0.35	0.15	0.16	92.43	100.12	109.76	14.52	14.44	15.95	3.41	3.55	2.55	96.83	96.26	106.30
Phenylalanine	2.5	2.43	2.68	2.74	1.18	0.88	1.22	97.21	107.14	109.59	2.39	2.82	2.70	2.05	5.96	1.59	95.68	112.77	107.89
	7.5	7.47	8.42	8.36	0.70	0.66	0.94	99.61	112.25	111.46	7.33	8.32	8.13	1.58	1.00	3.68	97.74	110.98	108.41
	15	14.20	16.58	16.97	0.70	0.59	0.48	94.66	110.53	113.15	14.55	15.78	16.79	1.91	4.78	0.93	96.99	105.21	111.92
Tryptophan	2.5	2.76	2.79	2.73	0.72	0.83	0.65	110.57	111.92	109.01	2.81	2.82	2.74	2.63	1.28	0.97	112.38	112.71	109.56
	7.5	8.60	8.30	7.38	0.39	0.99	1.74	114.70	110.66	98.42	8.51	8.27	7.40	0.82	2.79	2.29	113.52	110.31	98.68
	15	16.21	16.97	16.95	0.74	1.06	0.87	108.09	113.12	113.02	16.60	17.15	16.96	1.78	1.51	1.01	110.69	114.36	113.04
Tryptamine + 2-phenethyl-amine	2.5	2.50	2.58	2.49	1.07	0.41	0.69	100.20	103.05	99.79	2.50	2.58	2.50	1.11	1.22	0.75	100.03	103.13	99.99
	7.5	7.39	8.13	7.93	0.25	0.19	0.32	98.57	108.39	105.78	7.34	8.11	7.82	0.95	0.44	2.61	97.80	108.16	104.20
	15	14.03	15.96	16.30	0.11	0.26	0.21	93.52	106.42	108.66	14.36	15.35	16.24	1.85	4.24	0.35	95.73	102.35	108.23

Table S4: Recovery and matrix effect. QC: quality control.

Compound	QC concentration (µmol/L)	Mean concentration (µmol/L)			Recovery %			Matrix effect %	
		MQ	Serum	Tears	MQ	Serum	Tears	Serum	Tears
Histidine	2.5	2.80	2.82	2.78	112.18	112.83	111.15	25.80	-2.24
	7.5	8.13	7.99	8.12	108.41	106.41	108.25	8.86	2.77
	15	15.19	16.57	16.76	101.25	110.48	111.71	-6.59	-6.58
Asparagine	2.5	2.73	2.71	2.76	109.13	108.37	110.24	7.44	7.98
	7.5	8.20	8.49	8.36	109.32	113.26	111.50	0.08	2.90
	15	15.49	16.43	17.10	103.29	109.56	114.00	-3.06	-6.21
Taurine	2.5	2.40	2.73	2.40	95.89	109.36	96.00	6.00	2.47
	7.5	7.36	8.30	7.97	98.10	110.60	106.29	-3.86	-0.27
	15	13.96	16.09	16.21	93.05	107.24	108.09	-8.78	-6.29
Serine	2.5	2.57	2.68	2.56	102.84	107.09	102.27	9.42	-1.64
	7.5	7.74	8.36	8.33	103.19	111.47	111.04	-3.04	-0.51
	15	14.19	15.73	17.12	94.62	104.89	114.13	-7.79	-10.25
Glutamine	2.5	2.73	2.64	2.73	109.27	105.43	109.06	-22.04	-13.25
	7.5	8.47	8.08	8.00	112.87	107.72	106.72	2.48	2.48
	15	16.48	16.31	16.63	109.84	108.74	110.86	4.56	-1.44
Arginine	2.5	2.86	2.52	2.51	114.22	100.85	100.44	17.07	-8.06
	7.5	8.31	7.44	7.57	110.76	99.24	100.94	19.64	-2.35
	15	15.60	16.34	13.80	104.02	108.90	91.99	6.82	2.72
Histamine	2.5	2.25	2.61	2.41	89.82	104.26	96.26	16.35	2.87
	7.5	7.57	6.82	7.15	100.87	90.92	95.26	18.67	9.81
	15	13.47	14.41	13.71	89.77	96.03	91.39	-2.91	1.54
Glycine	2.5	2.41	2.74	2.67	96.25	109.67	106.76	6.64	1.49
	7.5	7.58	8.48	8.52	101.12	113.03	113.54	-4.78	-0.12
	15	14.23	16.15	16.98	94.84	107.64	113.20	-9.54	-6.47
Ethanolamine	2.5	2.55	2.72	2.73	101.81	108.84	109.18	11.01	-2.06
	7.5	7.53	8.21	8.21	100.34	109.50	109.41	-2.32	-1.20
	15	13.96	15.48	16.55	93.08	103.19	110.34	-6.62	-9.35
Aspartate	2.5	2.52	2.59	2.79	100.83	103.46	111.62	2.67	0.21
	7.5	7.90	8.42	8.07	105.28	112.27	107.60	-5.00	-0.03
	15	14.26	14.36	15.86	95.09	95.75	105.70	0.11	-10.98
Methylamine	2.5	2.66	2.79	2.73	106.28	111.55	109.06	2.47	3.45
	7.5	7.96	8.49	8.33	106.13	113.21	111.05	-4.62	-0.16
	15	14.48	14.58	16.83	96.56	97.22	112.17	0.23	-11.67
Glutamate	2.5	2.76	2.67	2.77	110.43	106.71	110.84	3.01	2.47
	7.5	8.22	8.46	8.27	109.56	112.84	110.24	-6.55	1.67
	15	15.07	14.96	16.78	100.45	99.73	111.87	-3.35	-8.97
Citrulline	2.5	2.67	2.79	2.78	106.61	111.50	111.17	11.58	1.49
	7.5	8.12	8.50	8.60	108.21	113.33	114.63	-0.54	1.12
	15	15.05	16.12	17.03	100.31	107.47	113.54	-5.69	-5.35
Threonine	2.5	2.60	2.67	2.64	103.95	106.65	105.41	9.73	-1.07
	7.5	7.73	8.50	8.40	103.02	113.31	111.99	-4.95	-0.77
	15	14.26	15.81	17.22	95.07	105.40	114.80	-7.48	-9.99
Alanine	2.5	2.68	2.71	2.72	107.17	108.53	108.93	-6.35	1.30
	7.5	7.96	8.17	8.47	106.18	108.95	112.86	-3.67	-1.21
	15	14.50	15.36	17.04	96.67	102.43	113.59	-6.09	-11.37
Ethylamine	2.5	2.61	2.76	2.82	104.50	110.33	112.98	3.77	3.99
	7.5	8.15	8.53	8.44	108.63	113.78	112.55	-2.28	0.90
	15	14.89	14.52	16.99	99.28	96.81	113.26	3.34	-10.99
Proline	2.5	2.53	2.80	2.55	101.26	112.11	102.13	1.44	0.58
	7.5	7.67	8.36	8.30	102.21	111.47	110.72	-4.51	-0.31
	15	14.25	15.65	17.05	95.02	104.35	113.64	-7.00	-9.12
Ornithine	2.5	2.70	2.82	2.78	108.15	112.89	111.22	4.54	0.79

	7.5	7.97	8.55	8.54	106.32	114.02	113.88	-5.21	-1.98
	15	14.39	15.04	17.01	95.94	100.26	113.43	-4.00	-12.21
Cysteine	2.5	2.56	2.81	2.63	102.51	112.52	105.32	7.14	0.14
	7.5	7.59	8.31	8.35	101.18	110.75	111.36	-1.59	0.37
	15	14.16	16.20	17.16	94.40	108.00	114.40	-8.48	-7.89
Lysine	2.5	2.79	2.75	2.80	111.50	109.86	111.81	7.76	17.14
	7.5	8.39	8.25	8.41	111.87	110.02	112.14	4.98	15.81
	15	15.16	16.11	16.94	101.07	107.41	112.96	-3.44	5.78
Tyrosine	2.5	2.49	2.72	2.64	99.44	108.79	105.61	13.94	12.59
	7.5	7.88	7.68	7.44	105.01	102.45	99.21	13.10	23.25
	15	15.40	15.92	16.00	102.68	106.12	106.67	4.88	15.85
Putrescine	2.5	2.80	2.82	2.70	111.89	112.97	107.82	8.67	3.27
	7.5	8.37	8.43	8.46	111.53	112.38	112.81	-2.45	-1.02
	15	15.21	14.97	17.14	101.40	99.77	114.26	-2.45	-12.42
Methionine	2.5	2.73	2.81	2.50	109.01	112.38	100.13	15.00	14.97
	7.5	8.17	8.03	8.29	108.96	107.08	110.56	10.38	12.56
	15	15.20	16.93	16.92	101.34	112.87	112.79	-4.34	5.64
Serotonin	2.5	2.37	2.85	2.48	94.73	114.12	99.22	18.20	-3.14
	7.5	6.71	7.60	7.48	89.52	101.34	99.78	0.99	0.13
	15	12.85	15.17	15.71	85.66	101.13	104.73	-9.76	-7.05
Valine	2.5	2.71	2.81	2.61	108.37	112.39	104.26	9.67	41.86
	7.5	8.37	8.20	8.29	111.55	109.36	110.50	11.20	40.64
	15	15.77	16.83	16.81	105.15	112.23	112.07	2.34	36.26
Cadaverine	2.5	2.79	2.74	2.76	111.39	109.57	110.22	13.49	20.26
	7.5	8.45	8.55	8.35	112.68	113.98	111.39	1.09	19.35
	15	15.36	15.69	17.02	102.39	104.63	113.45	-2.10	9.35
Tyramine	2.5	2.54	2.69	2.70	101.59	107.66	107.94	14.86	-1.77
	7.5	7.68	8.40	7.85	102.33	112.00	104.61	-1.35	-0.01
	15	14.64	16.61	15.79	97.61	110.71	105.27	-8.30	-6.08
Isoleucine	2.5	2.52	2.77	2.82	100.81	110.97	112.67	3.57	0.16
	7.5	7.54	8.27	8.14	100.58	110.23	108.48	-4.96	-1.45
	15	13.82	15.34	16.29	92.13	102.29	108.61	-8.44	-12.37
Leucine	2.5	2.74	2.67	2.75	109.68	106.67	109.99	-2.04	3.61
	7.5	7.73	8.26	8.39	103.09	110.17	111.81	-4.42	0.36
	15	13.87	15.02	16.46	92.43	100.12	109.76	-4.13	-7.91
Phenylalanine	2.5	2.43	2.68	2.74	97.21	107.14	109.59	13.18	-2.49
	7.5	7.47	8.42	8.36	99.61	112.25	111.46	-2.22	-0.30
	15	14.20	16.58	16.97	94.66	110.53	113.15	-9.07	-6.79
Tryptophan	2.5	2.76	2.80	2.73	110.57	111.92	109.01	21.87	0.67
	7.5	8.60	8.30	7.38	114.70	110.66	98.42	14.04	21.59
	15	16.21	16.97	16.95	108.09	113.12	113.02	3.57	6.09
Tryptamine + 2-phenethyl-amine	2.5	2.50	2.58	2.50	100.20	103.05	99.79	18.39	-0.57
	7.5	7.39	8.13	7.93	98.57	108.39	105.78	-0.17	0.96
	15	14.03	15.96	16.30	93.52	106.42	108.66	-6.54	-4.92

Table S5: Autosampler stability and freez-thaw stability. QC: quality control.

Compound	QC concentration (μmol/L)	Autosampler stability						Freeze-thaw cycle before derivatization						Freeze-thaw cycle after derivatization					
		Mean concentration (μmol/L)			Recovery%			Mean concentration (μmol/L)			Recovery%			Mean concentration (μmol/L)			Recovery%		
		MQ	Serum	Tears	MQ	Serum	Tears	MQ	Serum	Tears	MQ	Serum	Tears	MQ	Serum	Tears	MQ	Serum	Tears
Histidine	2.5	1.82	2.35	2.04	72.91	93.94	81.40	1.03	1.67	1.20	41.27	66.64	47.94	1.49	1.59	1.22	59.50	63.69	48.83
	7.5	6.65	7.39	5.81	88.65	98.58	77.41	5.41	4.13	5.31	72.16	55.02	70.76	5.13	5.88	5.20	68.38	78.40	69.31
	15	13.74	13.42	15.01	91.62	89.47	100.08	9.17	10.50	10.74	61.11	69.96	71.61	8.78	10.67	11.12	58.55	71.15	74.12
Asparagine	2.5	2.34	2.23	2.33	93.66	89.19	93.34	1.72	2.27	2.12	68.62	90.75	84.60	1.91	2.11	2.09	76.20	84.22	83.72
	7.5	6.98	6.76	6.80	93.04	90.17	90.72	6.69	5.78	6.84	89.20	77.06	91.15	5.61	6.05	6.04	74.79	80.67	80.56
	15	13.27	12.84	14.02	88.46	85.57	93.48	11.42	13.52	12.13	76.15	90.14	80.84	9.86	12.54	12.52	65.70	83.57	83.48
Taurine	2.5	2.31	2.68	2.28	92.20	106.98	91.10	1.87	2.65	1.93	74.87	105.99	77.33	2.12	2.44	2.03	84.95	97.41	81.19
	7.5	7.21	7.79	7.31	96.07	103.88	97.50	7.06	6.44	7.46	94.14	85.83	99.41	6.35	7.10	6.74	84.71	94.62	89.82
	15	14.00	13.98	15.75	93.36	93.21	105.02	12.14	14.47	13.15	80.96	96.44	87.65	11.07	13.85	14.25	73.82	92.35	94.97
Serine	2.5	2.41	2.64	2.17	96.44	105.45	86.78	1.71	2.57	1.85	68.46	102.68	74.11	2.09	2.34	1.87	83.54	93.61	74.69
	7.5	7.25	7.42	7.22	96.65	98.89	96.30	6.85	6.01	7.24	91.26	80.09	96.52	6.03	6.58	6.34	80.45	87.78	84.55
	15	13.86	13.50	15.62	92.39	89.99	104.16	11.63	13.67	12.76	77.55	91.16	85.09	10.04	12.82	13.32	66.92	85.48	88.79
Glutamine	2.5	2.22	1.97	1.92	88.64	78.76	76.65	0.84	1.57	0.86	33.61	62.78	34.31	1.31	1.30	0.88	52.30	52.05	35.38
	7.5	6.77	6.76	4.98	90.28	90.12	66.43	3.62	2.72	3.37	48.27	36.23	44.89	3.68	3.69	3.22	49.04	49.19	42.87
	15	12.63	11.70	13.10	84.20	77.99	87.34	4.70	5.60	6.36	31.35	39.91	42.37	4.48	5.53	5.50	29.88	36.83	36.67
Arginine	2.5	2.96	2.41	2.56	118.43	96.57	102.50	1.10	0.74	0.81	43.97	29.40	32.27	1.82	0.84	1.23	72.74	33.65	49.02
	7.5	8.38	9.01	5.76	111.78	120.12	76.76	3.41	1.81	2.69	45.45	24.12	35.85	4.73	4.89	4.05	63.03	65.20	53.94
	15	15.62	15.82	14.78	104.15	105.45	98.53	4.55	4.54	5.15	30.35	30.30	34.33	6.76	7.79	7.32	45.03	51.91	48.78
Histamine	2.5	2.37	2.85	2.32	94.90	114.09	92.97	1.17	1.90	1.50	46.91	76.11	60.09	1.85	2.12	1.81	73.89	84.64	72.36
	7.5	7.22	7.90	6.49	96.29	105.31	86.60	4.75	4.22	4.72	63.37	56.29	62.95	5.21	6.18	5.43	69.46	82.37	72.42
	15	14.08	13.91	14.69	93.84	92.76	97.95	7.80	9.15	8.35	51.96	60.99	55.66	8.78	10.95	10.88	58.56	72.97	72.50
Glycine	2.5	2.27	2.76	2.22	90.58	110.19	88.83	1.59	2.62	2.02	63.73	104.96	80.61	1.95	2.42	2.00	78.09	96.78	80.14
	7.5	7.27	7.80	7.39	96.91	104.01	98.58	6.85	6.18	7.34	91.34	82.35	97.82	5.90	6.77	6.55	78.72	90.20	87.32
	15	14.09	14.01	16.01	93.96	93.43	106.70	11.74	14.16	13.00	78.30	94.38	86.65	10.17	13.63	13.88	67.83	90.84	92.52
Ethanolamine	2.5	2.41	2.64	2.32	96.47	105.39	92.86	1.67	2.49	1.95	66.77	99.61	78.10	2.06	2.34	1.99	82.29	93.62	79.98
	7.5	7.50	7.66	7.53	100.04	102.16	100.38	7.16	6.25	7.51	95.44	83.36	100.08	6.18	6.85	6.45	82.40	91.28	85.98
	15	14.29	13.97	15.97	95.29	93.12	106.48	12.18	14.29	13.10	81.23	95.30	87.31	10.35	13.47	13.70	69.01	89.77	91.33
Aspartate	2.5	2.56	2.30	2.50	102.48	91.87	99.94	1.35	2.18	2.19	54.05	87.06	87.39	1.79	1.98	1.98	71.56	79.16	79.07
	7.5	8.15	7.32	7.60	108.62	97.59	101.30	7.31	6.14	7.49	97.47	81.87	99.85	6.18	6.26	6.03	82.38	83.53	80.35
	15	15.20	14.47	15.60	101.31	96.45	104.00	12.73	14.85	13.20	84.89	98.99	87.99	10.26	13.60	12.90	68.38	90.68	86.01
Methylamine	2.5	2.54	2.33	2.28	101.43	93.09	91.09	1.43	2.37	1.94	57.07	94.84	77.73	1.81	2.05	1.85	72.29	81.82	73.99
	7.5	8.01	7.28	7.52	106.78	97.10	100.24	7.28	6.09	7.31	97.01	81.22	97.51	5.48	6.01	5.82	73.07	80.07	77.62
	15	15.03	14.43	16.07	100.22	96.22	107.15	12.38	14.84	13.67	82.50	98.95	91.14	9.58	13.41	12.92	63.87	89.37	86.16

	2.5	2.71	2.32	2.45	108.36	92.94	97.79	1.68	2.22	2.05	67.27	88.88	81.94	2.04	2.01	2.08	81.73	80.38	83.19
Glutamate	7.5	8.18	7.23	7.57	109.03	96.37	100.96	7.10	5.56	7.15	94.70	74.15	95.27	6.17	6.11	6.21	82.29	81.54	82.74
	15	15.28	13.89	15.94	101.86	92.59	106.25	11.92	13.24	12.64	79.48	88.23	84.30	10.10	12.61	12.92	67.31	84.06	86.10
	2.5	2.49	2.73	2.48	99.65	109.23	99.54	1.71	2.49	2.02	68.22	99.59	80.90	2.14	2.40	2.16	85.73	95.78	86.24
Citrulline	7.5	7.48	7.65	7.49	99.67	101.99	99.90	6.73	5.79	7.09	89.71	77.22	94.57	6.26	6.70	6.59	83.46	89.37	87.82
	15	14.26	13.82	15.79	95.09	92.11	105.30	11.44	13.12	12.20	76.27	87.49	81.33	10.40	12.93	13.53	69.33	86.22	90.16
	2.5	2.43	2.56	2.17	97.09	102.37	86.63	1.76	2.50	1.98	70.20	100.09	79.09	2.05	2.35	1.90	81.82	94.11	76.15
Threonine	7.5	7.70	7.53	7.31	102.60	100.46	97.42	7.35	6.15	7.61	98.06	81.93	101.51	6.42	6.82	6.94	85.62	90.99	92.53
	15	15.02	14.58	16.86	100.14	97.18	112.39	12.91	14.80	14.08	86.09	98.67	93.85	10.39	13.59	14.41	69.25	90.61	96.08
	2.5	2.45	2.20	2.23	97.93	87.96	89.34	1.76	2.32	2.04	70.29	92.87	81.47	1.94	1.97	1.94	77.65	78.81	77.50
Alanine	7.5	7.44	6.74	6.93	99.16	89.90	92.40	7.39	6.24	7.64	98.51	83.13	101.88	5.58	5.89	5.76	74.40	78.50	76.84
	15	14.61	13.28	14.57	97.40	88.53	97.11	11.85	14.56	13.15	78.97	97.07	87.68	9.26	12.60	12.18	61.70	84.00	81.22
	2.5	2.50	2.27	2.32	100.05	90.63	92.82	1.35	2.24	1.91	54.08	89.69	76.55	1.62	1.96	1.88	64.87	78.37	75.21
Ethylamine	7.5	7.85	7.08	7.66	104.64	94.37	102.14	6.61	5.95	6.70	88.19	79.33	89.27	5.00	5.93	5.59	66.61	79.05	74.47
	15	14.35	14.07	15.63	95.70	93.78	104.17	11.30	14.35	12.97	75.36	95.67	86.47	9.53	13.35	11.80	63.54	89.00	78.68
	2.5	2.38	2.51	2.19	95.18	100.47	87.75	1.74	2.52	1.91	69.44	100.77	76.23	2.00	2.30	1.89	80.00	91.84	75.60
Proline	7.5	7.36	7.31	7.51	98.07	97.48	100.17	7.19	6.15	7.25	95.87	81.98	96.67	6.15	6.75	6.28	82.04	89.96	83.67
	15	14.13	13.46	15.57	94.19	89.75	103.82	11.65	13.72	13.36	77.67	91.48	89.07	9.97	13.30	13.75	66.44	88.64	91.63
	2.5	2.58	2.29	2.16	103.11	91.82	86.44	1.63	2.53	2.10	65.06	101.17	83.88	1.85	2.09	1.90	74.08	83.60	75.99
Ornithine	7.5	7.17	6.72	7.03	95.62	89.53	93.66	7.43	6.22	7.68	99.00	82.91	102.35	5.45	5.98	5.63	72.66	79.79	75.08
	15	14.88	12.82	14.91	99.23	85.47	99.43	12.75	14.56	13.31	84.99	97.05	88.75	9.49	12.34	12.25	63.27	82.28	81.63
	2.5	2.25	2.53	2.16	90.18	101.13	86.47	1.73	2.51	1.92	69.08	100.46	76.87	1.98	2.31	1.86	79.12	92.48	74.49
Cysteine	7.5	6.73	7.22	6.98	89.70	96.31	93.12	6.59	5.88	7.15	87.91	78.44	95.35	5.96	6.64	6.47	79.51	88.56	86.24
	15	13.28	13.11	15.39	88.53	87.42	102.59	11.41	13.58	12.29	76.08	90.54	81.93	10.33	12.87	13.75	68.87	85.77	91.65
	2.5	2.47	2.29	2.55	98.85	91.57	102.04	1.41	2.23	2.14	56.47	89.00	85.76	1.84	2.11	2.25	73.43	84.28	89.91
Lysine	7.5	7.50	6.92	8.06	100.00	92.26	107.50	6.53	6.82	7.63	86.99	90.91	101.71	5.55	6.00	6.52	73.93	80.00	86.98
	15	13.92	13.52	16.99	92.79	90.15	113.24	10.95	10.28	13.43	73.00	68.52	89.54	9.24	12.75	13.95	61.61	84.97	92.98
	2.5	2.37	2.78	2.77	94.80	111.09	110.90	1.73	2.38	2.14	69.38	95.26	85.60	2.24	2.57	2.56	89.78	102.84	102.38
Tyrosine	7.5	7.03	7.70	8.29	93.70	102.70	110.51	6.07	5.70	7.30	80.89	75.98	97.34	6.39	7.30	7.76	85.21	97.27	103.51
	15	13.61	13.68	17.36	90.73	91.19	115.75	10.70	12.33	12.66	71.32	82.17	84.40	11.53	14.06	16.22	76.89	93.74	108.10
	2.5	2.61	2.50	2.20	104.24	100.06	88.13	1.56	2.45	1.88	62.52	97.82	75.23	1.88	2.20	1.84	75.04	87.81	73.63
Putrescine	7.5	8.04	7.35	7.30	107.21	98.05	97.32	6.97	5.62	6.76	92.87	74.97	90.17	5.60	6.05	5.51	74.61	80.64	73.46
	15	15.23	13.96	15.61	101.56	93.08	104.09	11.69	13.30	11.62	77.96	88.67	77.46	9.34	12.52	11.93	62.24	83.48	79.51
	2.5	2.37	2.62	2.37	94.93	104.58	94.66	1.74	2.49	1.98	69.45	99.73	79.15	2.10	2.40	2.09	84.17	95.80	83.60
Methionine	7.5	7.03	7.45	7.62	93.77	99.30	101.63	6.55	5.92	7.42	87.33	78.91	98.94	6.12	6.82	6.94	81.66	90.95	92.55
	15	13.52	13.48	16.31	90.15	89.87	108.75	11.23	13.29	12.90	74.88	88.57	85.98	10.54	13.32	14.71	70.26	88.83	98.07
Serotonin	2.5	2.28	2.94	2.20	91.29	117.66	88.06	2.00	2.87	1.95	79.95	114.70	78.07	2.34	2.80	2.14	93.57	111.94	85.65
	7.5	6.27	7.53	7.00	83.57	100.38	93.36	6.27	6.34	7.03	83.64	84.52	93.71	6.09	7.24	6.85	81.16	96.52	91.39

	15	12.62	13.05	15.63	84.11	86.99	104.20	10.94	13.28	12.42	72.94	88.56	82.80	11.13	13.76	14.89	74.16	91.73	99.27
Valine	2.5	2.34	2.55	3.49	93.65	101.92	139.75	1.03	1.47	1.79	41.30	58.88	71.77	2.04	2.38	3.26	81.42	95.34	130.49
	7.5	7.05	7.43	10.74	93.97	99.08	143.23	4.04	3.41	6.16	53.88	45.48	82.10	6.03	6.86	9.84	80.38	91.51	131.14
	15	13.46	13.74	22.69	89.73	91.61	151.25	6.92	7.71	10.57	46.11	51.41	70.45	10.17	13.70	20.54	67.80	91.32	136.93
Cadaverine	2.5	2.61	2.45	2.65	104.22	97.92	106.12	1.16	1.91	1.81	46.47	76.47	72.27	1.86	2.23	2.34	74.25	89.13	93.63
	7.5	7.76	7.08	8.47	103.45	94.41	112.92	5.57	4.37	6.37	74.22	58.20	84.99	5.51	6.03	6.67	73.42	80.35	88.88
	15	14.37	13.67	17.92	95.80	91.12	119.48	9.32	10.58	11.28	62.12	70.54	75.20	9.34	12.72	14.46	62.24	84.78	96.42
Tyramine	2.5	2.49	2.85	2.48	99.51	113.96	99.24	2.04	2.80	2.23	81.53	111.94	89.27	2.32	2.57	2.30	92.83	102.92	91.88
	7.5	7.29	7.82	7.17	97.21	104.31	95.53	7.38	6.84	7.42	98.45	91.13	98.96	6.42	7.19	6.55	85.60	95.92	87.32
	15	14.08	13.95	14.86	93.87	92.97	99.09	12.89	15.06	12.81	85.96	100.39	85.41	11.57	14.05	13.70	77.13	93.64	91.31
Isoleucine	2.5	2.45	2.56	2.52	98.00	102.22	100.61	1.74	2.54	2.23	69.65	101.55	89.12	2.06	2.33	2.28	82.56	93.15	91.07
	7.5	7.32	7.33	7.23	97.64	97.71	96.43	6.93	6.05	7.24	92.45	80.69	96.58	6.03	6.57	6.38	80.44	87.59	85.07
	15	13.99	13.54	15.04	93.25	90.23	100.27	11.81	13.87	12.39	78.73	92.49	82.61	10.24	13.14	13.12	68.28	87.58	87.46
Leucine	2.5	2.74	2.30	2.44	109.63	92.15	97.59	1.73	1.84	1.57	69.14	73.68	62.92	2.15	2.06	2.20	86.07	82.47	87.96
	7.5	7.84	7.35	7.64	104.48	98.00	101.91	5.87	5.44	4.94	78.24	72.55	65.92	6.11	6.45	6.52	81.46	86.05	86.99
	15	14.14	13.89	15.46	94.26	92.58	103.09	10.76	13.93	9.66	71.74	92.84	64.43	10.18	13.34	13.66	67.83	88.92	91.07
Phenylalanine	2.5	2.28	2.72	2.42	91.23	108.90	96.71	1.83	2.59	2.11	73.38	103.51	84.45	2.20	2.51	2.28	87.99	100.20	91.25
	7.5	6.80	7.50	7.28	90.71	100.04	97.12	6.66	6.27	7.32	88.85	83.62	97.55	6.26	7.07	6.91	83.49	94.23	92.16
	15	13.20	13.36	15.30	87.97	89.03	102.01	11.70	13.84	12.78	78.02	92.25	85.20	11.28	13.70	14.51	75.21	91.33	96.70
Tryptophan	2.5	2.42	2.80	2.22	96.85	112.12	88.91	2.07	2.73	1.92	82.70	109.19	76.69	2.25	2.63	2.10	90.16	105.24	84.00
	7.5	6.62	7.30	6.80	88.27	97.40	90.64	6.43	6.37	6.81	85.67	84.88	90.81	6.12	6.99	6.51	81.65	93.17	86.79
	15	12.55	12.80	14.31	83.67	85.31	95.39	11.21	13.44	12.04	74.75	89.57	80.24	11.13	13.36	13.89	74.20	89.09	92.60
Tryptamine + 2-phenethyl-amine	2.5	2.19	2.55	2.00	87.76	101.83	79.85	1.78	2.47	1.71	71.14	98.71	68.46	2.04	2.32	1.84	81.43	92.94	73.49
	7.5	6.24	6.83	6.39	83.21	91.10	85.26	6.12	5.83	6.44	81.62	77.76	85.81	5.57	6.37	5.88	74.21	84.88	78.45
	15	13.94	13.96	13.59	92.90	93.04	90.60	10.60	12.62	11.32	70.67	84.13	75.45	11.74	14.32	12.72	78.24	95.47	84.82
